# Multilocus Sequence Typing of Non-JP2 Serotype b *Aggregatibacter actinomycetemcomitans* Strains of Ghanaian and Swedish Origin

**DOI:** 10.3389/fcimb.2021.769671

**Published:** 2021-12-14

**Authors:** Rolf Claesson, Anders Johansson, Carola Höglund Åberg, Anders Esberg, Dorte Haubek, Jan Oscarsson

**Affiliations:** ^1^ Division of Oral Microbiology, Department of Odontology, Umeå University, Umeå, Sweden; ^2^ Division of Molecular Periodontology, Department of Odontology, Umeå University, Umeå, Sweden; ^3^ Department of Odontology, Umeå University, Umeå, Sweden; ^4^ Section for Paediatric Dentistry, Department of Dentistry and Oral Health, Aarhus University, Aarhus, Denmark

**Keywords:** *Aggregatibacter actinomycetemcomitans*, multilocus sequence typing (MLST), non-JP2, serotype b, *cagE*, evolutionary analysis, genomic analysis

## Abstract

**Objective and Methods:**

The Gram-negative bacterium, *Aggregatibacter actinomycetemcomitans* is associated with periodontitis affecting young individuals. The geographic dissemination of the highly leukotoxic JP2 genotype of serotype b of this species was previously studied by multilocus sequence typing (MLST). Here, we have used MLST to genetically characterize non-JP2 genotype strains of serotype b, isolated from individuals living in Ghana (n=41), and in Sweden (n=13), respectively.

**Results:**

The MLST analysis revealed a total of nine sequence types (ST). Both Ghanaian and Swedish isolates were distributed in ST 1-3. ST 5 and 6 were only identified among the Ghanaian strains, whereas ST 4, 7, 8 and 9 were uniquely represented among the Swedish strains. Previously, we characterized these non-JP2 genotype strains of *A. actinomycetemcomitans* serotype b by arbitrarily-primed (AP)-PCR, which distributed them into three groups, AP-PCR type 1, 2, and 3, respectively. AP-PCR type 1 strains are generally highly leukotoxic, and are associated with progression of periodontal attachment loss. As AP-PCR type 1 includes both JP2 genotype strains and a proportion of non-JP2 genotype strains of serotype b, a straightforward diagnostic procedure has been sought. This has revealed a gene, *cagE*, which appears to be conserved only in this AP-PCR type. According to our results, MLST was not a highly discriminatory method to identify AP-PCR type 1, as strains of this AP-PCR type could be found within three different ST: ST 2, ST 3 and ST 8.

**Conclusion:**

According to MLST, a geographic dissemination of non-JP2 genotype *A. actinomycetemcomitans* serotype b appears to exist. However, aiming to identify carriers of AP-PCR type 1, non-JP2 genotype serotype b, PCR with *cagE*-specific primers is likely the most efficient diagnostic procedure known today.

## Introduction

*Aggregatibacter actinomycetemcomitans* produces a toxin, which associates this bacterium with periodontitis affecting young individuals (Haubek et al., 2008; Henderson et al., 2010; Könönen and Muller, 2014; Fine et al., 2020). Since the toxin causes lethal effects on leukocytes of different types, it is considered a leukotoxin (LtxA). The production and release of LtxA is affected by alterations in the promoter region of the *ltxCABD* operon (Kolodrubetz et al., 1989; Spitznagel et al., 1991). Of the seven serotypes of *A. actinomycetemcomitans* (a-g), a specific variant of serotype b has a deletion of 530 base pairs (bp) in the promoter region (Brogan et al., 1994). This variant is genetically different from those having a full-length promoter, and is since long referred to as the JP2 genotype (Haubek, 2010). As a consequence, all variants of *A. actinomycetemcomitans* with a full-length leukotoxin gene promoter are typically referred to as non-JP2 genotype. In serotype b strains, also additional *ltxCABD* promoter types have been identified, with either deletions of alternative regions of the promoter, or with insertions in it (He et al., 1999; Claesson et al., 2015; Claesson et al., 2020).

The JP2 genotype of *A. actinomycetemcomitans* serotype b is highly leukotoxic (Tsai et al., 2018), and carriers of this specific type are at substantial risk for rapid loss of tooth-supporting tissues, including alveolar bone (Bueno et al., 1998; Haraszthy et al., 2000; Haubek et al., 2008; Burgess et al., 2017). While the JP2 genotype appears to be distributed only in serotype b of *A. actinomycetemcomitans*, the non-JP2 genotype is found among all serotypes (a-g). Non-JP2 genotype strains of *A. actinomycetemcomitans* are generally low-leukotoxic. However, a subgroup of non-JP2 genotype strains of *A. actinomycetemcomitans* serotype b that exhibit high leukotoxicity, and has enhanced association with periodontal disease progression, has been identified (Asikainen et al., 1995; Höglund Åberg et al., 2014).

In addition to leukotoxin promoter typing, *A. actinomycetemcomitans* strains have been genotypically characterized by a range of other DNA-based methods (Asikainen and Karched, 2008; Kittichotirat et al., 2016). One of them is arbitrarily-primed (AP)-PCR (van Belkum, 1994), which has revealed that all hitherto tested JP2 genotype strains belong to the same AP-PCR banding pattern group (AP-PCR type 1), whereas for non-JP2 strains a number of different AP-PCR types have been identified (Höglund Åberg et al., 2014; Claesson et al., 2017). Interestingly, a subgroup of non-JP2 genotype strains of *A. actinomycetemcomitans* serotype b that expresses enhanced levels of LtxA also belongs to AP-PCR type 1, and hence appears to be genetically related to the JP2 genotype of *A. actinomycetemcomitans* (Höglund Åberg et al., 2014; Johansson et al., 2017). Moreover, all hitherto assessed *A. actinomycetemcomitans* strains of serotype b belonging to AP-PCR type 1 have the property in common that they carry the gene *cagE*, which with very few exceptions appears to be present in this AP-PCR type only, and as the gene is absent in the other *A. actinomycetemcomitans* serotypes, it could be used for diagnostic purposes (Johansson et al., 2017; Johansson et al., 2019).

AP-PCR characterization mirrors genetic variability within bacterial species without requiring the analysis of specific genes, whereas Multilocus Sequence Typing (MLST) is a molecular typing method that is used to characterize bacterial strains in terms of pathogenicity and dissemination (Dogan et al., 1999b; Haubek et al., 2007; Jolley and Maiden, 2014). This is a highly discriminatory typing method based on sequencing of a number of housekeeping genes. MLST of bacterial strains renders so called sequence types (ST), which can be further used in dissemination studies. As judged by the MLST-based characterization, the JP2 genotype of serotype b emerged from the Mediterranean part of Africa about 2400 years ago, and has then subsequently been spread worldwide (Haubek et al., 2007; Rylev and Kilian, 2008).

In the present study, we have carried out MLST-characterization of non-JP2 genotype strains of *A. actinomycetemcomitans* serotype b from two of our clinical collections, isolated from individuals living in Ghana and Sweden, respectively (Höglund Åberg et al., 2012; Claesson et al., 2017). Thereby, we have been able to compare the genetic variability of non-JP2 genotype strains of *A. actinomycetemcomitans* serotype b collected from carriers living in two from each other separated continents, to assess if geographic dissemination of such strains may exist. In addition, our aim was to study whether any correlation could be observed between AP-PCR/*cagE* genotype and MLST–based sequence types of non-JP2 genotype strains of *A. actinomycetemcomitans* serotype b. To this end, the MLST pattern of each strain was compared with their genetic discrepancies earlier revealed by AP-PCR and *cagE* genotyping (Höglund Åberg et al., 2014; Claesson et al., 2017; Johansson et al., 2017; Johansson et al., 2019).

## Materials and Methods

### *A. actinomycetemcomitans* Strain Collections

A list of all strains used in this work is presented as **Supplementary Material** (**Table S1**). A total of 56 non-JP2 genotype strains of *A. actinomycetemcomitans* serotype b, collected and isolated from subgingival plaque samples, were used in this work. These included 41 collected from adolescents in a Ghanaian (Gh) study cohort (Höglund Åberg et al., 2012), 13 sampled from patients living in Sweden (Sw), and recruited to specialist periodontal treatment (Claesson et al., 2017), and two reference strains named HK908, and HK912. For all strains used in this work, AP-PCR and *cagE* genotype have earlier been examined, respectively (Höglund Åberg et al., 2014; Claesson et al., 2017; Johansson et al., 2017; Johansson et al., 2019). The 56 strains were grouped into AP-PCR type 1 (n=18, all *cagE*-positive), AP-PCR type 2 (n=16; all *cagE*-negative), and AP-PCR type 3 (n=21; 20 *cagE*-negative). The latter AP-PCR type actually includes AP-PCR types 3 to 11, as defined earlier (Claesson et al., 2017; Johansson et al., 2019). One strain, Gh 26 (*cagE*-negative), was non-typeable by AP-PCR. Of the strains isolated in Ghana, 15 are AP-PCR type 1, 13 type 2, and 12 type 3. Of the strains isolated in Sweden, two are AP-PCR type 1, two are type 2 and nine type 3. Moreover, *A. actinomycetemcomitans* strains HK908 (AP-PCR type 2), originally referred to as non-serotypeable (Haubek et al., 2007), but later confirmed as serotype b (Johansson et al., 2017), and HK912 (AP-PCR type 1; serotype b) (Haubek et al., 2007) were used as references. All strains were cultured on blood agar plates (5% defibrinated horse blood, 5 mg hemin/L, 10 mg vitamin K/L, Columbia agar base) and incubated in air supplemented with 5% CO_2_, at 37°C.

### Multilocus Sequence Typing (MLST) and Evolutionary Analyses

The gene fragments used for MLST in this study were originally selected from the *A. actinomycetemcomitans* serotype b, JP2 genotype strain, named HK1651 (Haubek et al., 2006), which were utilized for the analysis of JP2 genotype strains (Haubek et al., 2007). In the present study, fragments of genes encoding four housekeeping enzymes were selected for MLST: *recA* (GenBank accession EF142768) encoding the RecA protein, *adk* (EF142164) encoding adenylate kinase, *frdB* (EF142336) encoding fumarate reductase, and *atpG* (EF142218) encoding the gamma subunit of ATP synthase F1. In addition, two fragments of the hemoglobin-binding protein pseudogene *hbpA* (*hbpA-1*, and *hbpA-2*; EF142408 and EF142489), and one fragment of the transferring-binding protein pseudogene, *tbpA* (EF142817). Templates for PCR were obtained by boiling a loopful of *A. actinomycetemcomitans* colonies in water. PCR amplification and DNA sequencing were performed as previously described (Haubek et al., 2007). The MLST sequence types (ST) of the 56 non-JP2 genotype strains were defined based on single nucleotide polymorphisms (SNPs) within the seven DNA fragments. A cluster analysis of the 56 *A. actinomycetemcomitans* strains was performed based on the concatenated sequences of the seven DNA fragments in the order *adk, atpG, frdB, recA, hbpA*-*1, hbpA*-*2, and tbpA*. Evolutionary analyses were conducted by using the Minimum Evolution algorithm in MEGA, version 7.21 (Tamura et al., 2013). A total of 3176 nucleotide positions were used in the final dataset.

### Ethical Considerations

Ethical clearance for this work was obtained from the Noguchi Memorial Institute for Medical Research, University of Ghana (IRB 000 1276), and from the local ethical committee of Umeå University, Sweden (Dnr 2010-188-31M).

## Results

### Identification of Polymorphic Sites and SNPs

The MLST analysis of a total of 56 non-JP2 genotype strains of *A. actinomycetemcomitans* serotype b, including the reference strains HK908 and HK912, was based on four house-keeping genes and three additional gene fragments, as described in Materials and Methods. When using the JP2 genotype strain, HK1651 as a reference for the comparisons, 14 polymorphic sites could be identified (**Table 1**). However, four polymorphic sites, located in the *atpG* (at nucleotide [nt] position 231), *hbpA-1* (at nt 44), and in the *hbpA-2* (at nt 6 and nt 214) gene fragments, respectively, were unique for HK1651, and there was no variation among the 56 non-JP2 genotype strains at these sites. In these strains, on the other hand, six polymorphic sites were identified in one strain each: *adk* (at nt 107), *atpG* (at nt 459 and 479), *frdB* (at nt 143), *recA* (at nt 111), and *hbpA-2* (at nt 123). In all of the 56 non-JP2 genotype strains, a total of 290 SNPs were detected at the ten polymorphic sites, representing ≈0.1% of the total number of nucleotides of the sequenced gene fragments.

**Table 1:** The polymorphic sites of the nine sequence types (ST) identified by MLST analysis of 56 non-JP2 genotype, serotype b *A. actinomycetemcomitans* strains.

ST																		
		Gh	Sw	Ref	*adk*	*atpG*				*frdB*		*recA*	*hbpA-1*		*hbpA-2*			*tbpA*
	lenght (bp)				559	500				510		495	439		329			344
																		
	position				107	231	318	459	479	143	272	111	44	307	6	123	214	56
																		
1		13	1		C	**C**	G	C	G	G	G	C	**G**	**T**	**G**	G	**G**	-
2		21	4	1(a)	C	**C**	G	C	G	G	G	C	**G**	C	**G**	G	**G**	-
3		5	3	1(b)	C	**C**	G	C	G	G	G	C	**G**	C	**G**	G	**G**	A
4			2		C	**C**	**A**	C	G	G	G	C	**G**	C	**G**	G	**G**	A
5		1			C	**C**	G	C	G	**A**	G	C	**G**	C	**G**	G	**G**	-
6		1			C	**C**	G	**T**	**T**	G	G	C	**G**	C	**G**	G	**G**	A
7			1		C	**C**	G	C	G	G	**T**	C	**G**	C	**G**	G	**G**	A
8			1		C	**C**	G	C	G	G	G	**A**	**G**	C	**G**	G	**G**	-
9			1		**T**	**C**	G	C	G	G	**T**	C	**G**	C	**G**	**T**	**G**	A
																		
		41	13	2														
																		
HK1651					*C*	*G*	*G*	*C*	*G*	*G*	*G*	*C*	*T*	*C*	*T*	*G*	*A*	*A*

Indicated are the polymorphic sites identified in the gene fragments adk, atpG, frdB, recA, hbpA-1, hbpA-2 and tbpA, which were used for MLST analysis 56 non-JP2 serotype b strains of Ghanaian (Gh) and Swedish (Sw) origin, respectively. The reference non-JP2 serotype b strains (Ref; HK912 [a], and HK908 [b]) are also included in the table. For comparison, the polymorphic sites of A. actinomycetemcomitans strain, HK1651 a JP2 genotype reference strain (Haubek et al., 2006; Haubek et al., 2007), is included in the table. Bold letters in the table show nucleotides that are distinguished from HK1651.

### Identification of Sequence Types

Based on the MLST scheme used, nine sequence types (ST) could be identified among the 56 non-JP2 genotype strains of *A. actinomycetemcomitans* serotype b, and which in this work were denoted ST 1 to ST 9 (**Table 2**). The two non-JP2 genotype serotype b reference strains, HK908 and HK912, were confirmed to belong to ST 3 and ST 2, respectively. The overall most common sequence types among the 56 strains were ST 1 (25%), and ST 2 (46.3%). The number of SNPs among the different ST varied between four and seven (**Table 1**).

**Table 2:** Sequence types (ST) of the 56 non-JP2 serotype b *A. actinomycetemcomitans* strains.

ST	1	2	3	4	5	6	7	8	9	n
Gh	13	21	5		1	1				41
Sw	1	4	3	2			1	1	1	13
Ref		1(a)	1(b)							2
Total	14	26	9	2	1	1	1	1	1	56
%	25	46.3	16.1	3.6	1.8	1.8	1.8	1.8	1.8	100

The numbers of Ghanaian (Gh), and Swedish (Sw) serotype b A. actinomycetemcomitans non-JP2 genotype strains distributed according to the nine detected sequence types (ST 1 to 9), and the proportions of the different ST are indicated. The reference strains (Ref; HK912 [a], and HK908 [b]) are also included in the table.

### Distribution of Sequence Types Among the Strains Isolated in Ghana and Sweden and Cluster Analysis

**Figure 1:**
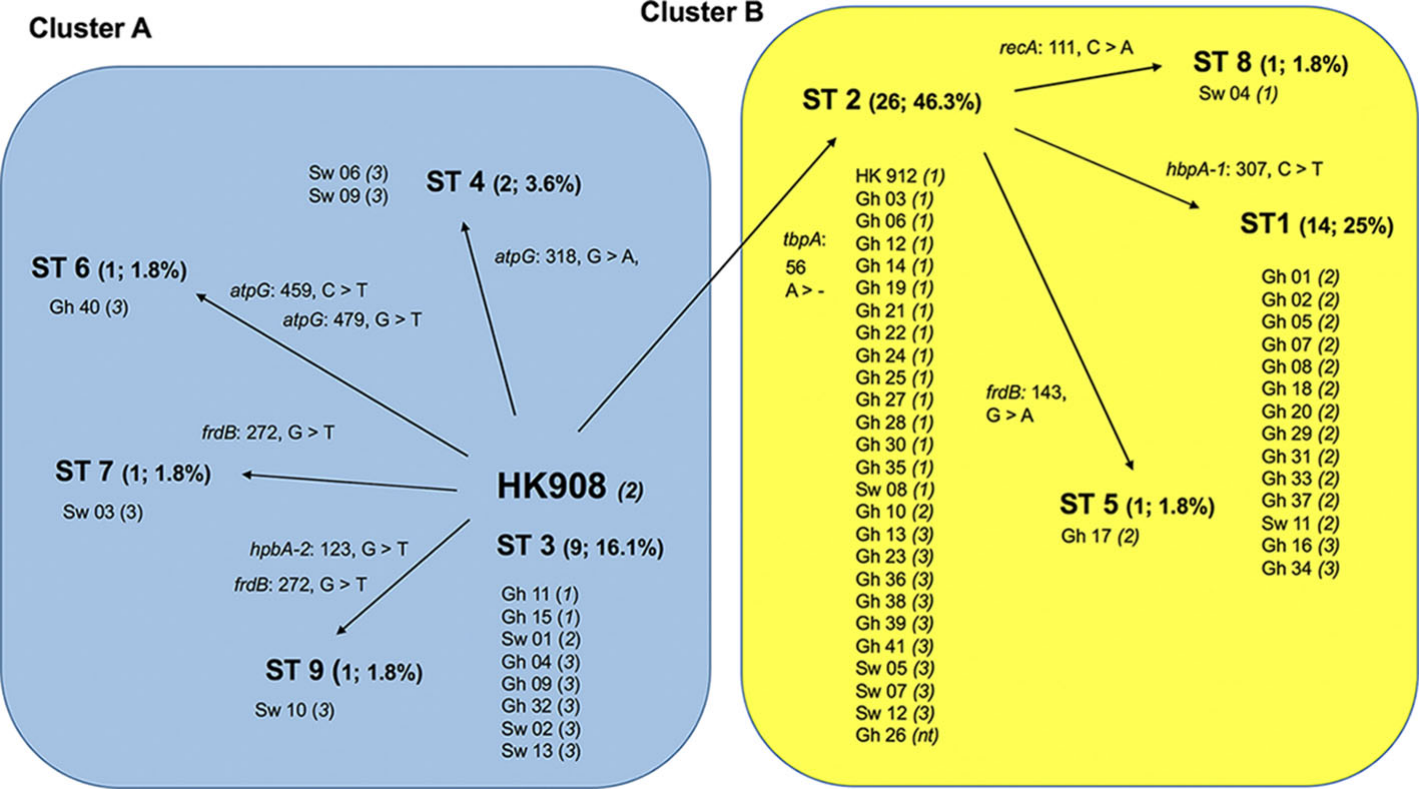
Schematic illustration of the separation of 56 non-JP2 genotype strains of *A. actinomycetemcomitans*, serotype b strains into two clusters by MLST. Cluster A (blue background), and B (yellow), respectively, were separated based on the nine identified sequence types (ST 1-9). The numbers and proportions of each ST out of the 56 strains are indicated. All ST distributed into Cluster B carry the deletion of an “A” at nucleotide position 56 in *tbpA* (tbpA 56 >-). The AP-PCR-type (1-3, or non-typeable [nt]) is indicated within parentheses for each strain. Gh, Ghanaian strains; Sw, strains of Swedish origin. The reference strains HK908 and HK912 are included in the diagram.

**Figure 2:**
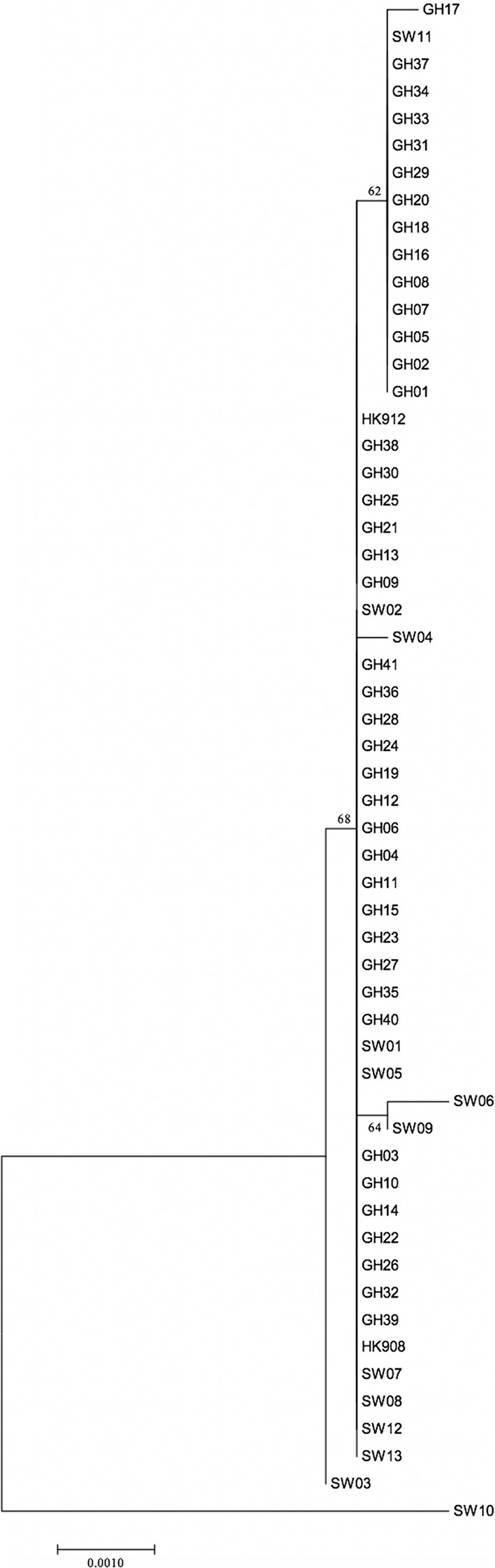
Phylogenetic relationship of 56 non-JP2 genotype *A. actinomycetemcomitans* strains of serotype b, based on MLST. The phylogenetic tree with the highest log likelihood is shown. The percentages of trees, in which the associated strains clustered together, is shown next to the branches. The tree is drawn to scale, with branch lengths measured in the number of substitutions per site. The bar indicates genetic distance. GH, Ghanaian strains; SW, Swedish strains.

As indicated in **Table 2**, a large portion of the non-JP2 genotype strains of *A. actinomycetemcomitans* of serotype b isolated in Ghana and Sweden, respectively, were distributed in ST 1 to 3, which were the only sequence types identified in both countries. This supports that geographic dissemination of the non-JP2 genotype of *A. actinomycetemcomitans* serotype b may exist. In both collections of non-JP2 genotype serotype b strains, ST2 was the most common, representing 51.2% of the Ghanaian and 30.8% of the Swedish strains, respectively. The second most frequent sequence type, ST 1, was more common among the strains of Ghanaian (n=13) relative to those of Swedish origin (n=1). Moreover, whereas ST 5 and 6 were only identified among the Ghanaian strains, ST 4, 7, 8, and 9 were unique for the strains isolated in Sweden (**Table 2**). Two major clusters could be identified based on the presence and absence of a deletion of an “A” at nt position 56 in *tbpA*, respectively (**Figure 1**). The cluster lacking this deletion, referred to as cluster A, contained 14 strains, which were distributed in ST 3, 4, 6, 7, and 9. Cluster B contained 42 strains with the deletion in *tbpA*, i.e., these strains exhibited a deleted “A” at nt position 56 in *tbpA* (ST 1, 2, 5 and 8). Among the 41 Ghanaian strains, 35 (85%) were distributed in cluster B, consistent with a rather homogeneous population of non-JP2 genotype strains of serotype b. In contrast, the strains of Swedish origin appeared to be more heterogeneous, with seven (54%) belonging to cluster A, and the remaining 6 (46%) to cluster B. This difference may reflect that there is a diversity in the genetic composition between the strains of Ghanaian and Swedish origin. However, as shown in the phylogenetic tree (**Figure 2**), cluster analysis, using the seven concatenated gene sequences revealed a close genetic relationship between essentially all 56 strains regardless of country of origin. We observed though that all 14 ST 1 strains in cluster B were represented in a separate monophyletic branch, and that strain Sw 10 (ST 9) appeared more distantly related.

### Correlation Between MLST-Based Sequence Types and AP-PCR Genotype

The 56 non-JP2 genotype strains of *A. actinomycetemcomitans*, serotype b strains were earlier assessed by AP-PCR, and were distributed into AP-PCR types 1, 2 and 3, as described in Materials and Methods. The AP-PCR type of each of the nine MLST-based ST found among the Ghanaian and Swedish strains together, including the reference strains HK908 and HK912, is indicated in **Table 3**. Among the 18 AP-PCR type 1 (and *cagE^+^*) strains, a large majority, i.e., 14 (77.8%) were found to belong to ST 2. However, ST 2 did not exhibit any unique SNP at the ten polymorphic sites (**Table 1**). Of the 16 AP-PCR type 2 strains, the majority (12; 75%) belonged to ST 1. Thus, the polymorphic site at nt 307 in *hbpA-1* (**Table 1**), unique for ST 1 strains, could be considered rather strongly associated with AP-PCR type 2. The 21 AP-PCR type 3 strains, were as expected a less homogeneous group, and were distributed into ST 1, 2, 3, 4, 6, 7, and 9. Based on these observations together, we could conclude that the AP-PCR genotypes to some extent do cluster into specific MLST-defined ST, but that *cagE*-genotyping presently appears to be a stronger, and more easily implemented tool to diagnose carriage of virulent non-JP2 genotype strains of *A. actinomycetemcomitans* serotype b.

**Table 3:** AP-PCR types of the 56 non-JP2 serotype b *A. actinomycetemcomitans* strains.

ST	Sw				Gh					Ref		
		AP1	AP2	AP3		AP1	AP2	AP3	nt		AP1	AP2
1	**1**		1		**13**		11	2				
2	**4**	1		3	**21**	13	1	6	1	**1**	1(a)	
3	**3**		1	2	**5**	2		3		**1**		1(b)
4	**2**			2	**0**							
5	**0**				**1**		1					
6	**0**				**1**			1				
7	**1**			1								
8	**1**	1										
9	**1**			1								
Total	**13**	2	2	9	**41**	15	13	12	1	**2**	1	1

Indicated is the distribution of AP-PCR types within the different ST of the non-JP2 genotype strains of A. actinomycetemcomitans, serotype b of Ghanaian (Gh) and Swedish (Sw) origin. The reference (Ref) strains, HK912 (a) and HK 908 (b), which were distributed in AP-PCR type 1 and 2, respectively, are also included. One Ghanaian strain was non-typeable (nt) by AP-PCR. Totals are highlighted in bold.

## Discussion

In this work, we have used MLST to investigate the genetic diversity of 56 non-JP2 genotype strains of *A. actinomycetemcomitans* serotype b, with 41 strains originating from Ghana and 13 from Sweden.

Interestingly, serotype b includes a genetic variant that can be found both among JP2 genotype, and among some non-JP2 genotype strains. We have earlier demonstrated that these strains belong to the same AP-PCR type (i.e., AP-PCR type 1) (Johansson et al., 2017). While JP2 genotype strains of *A. actinomycetemcomitans* previously have been characterized by MLST, corresponding studies of non-JP2 strains of serotype b have been scarcely reported on (Haubek et al., 2007). Based on the MLST analysis of the 56 non-JP2 genotype serotype b strains, we suggest that the overall mutation rate of this genotype is rather low. Comparing the nine identified MLST sequence types with their corresponding AP-PCR genotype, we found both similarities and discrepancies, as the strains were distributed in different clusters based on the two genotyping methods.

In this study, although the mutation rate within the non-JP2 genotype strains appeared to be low as evidenced by MLST, discrepancies between the strains of Ghanaian and Swedish origin were detected. This was emphasized by the five unique SNPs among the Ghanaian strains, and seven among the strains isolated from individuals living in Sweden, despite that the number of assessed Swedish strains was more than three times lower. This indicates that the Swedish strains may represent a more diverse group. The reason for this could probably be that the Ghanaian collection of strains was sampled from a more homogeneous group of individuals with regards to age and geographic localization (Höglund Åberg et al., 2012). The notion that *A. actinomycetemcomitans* serotype b that colonizes the Swedish population, are more prone to mutations is another possible explanation.

*A. actinomycetemcomitans* strain HK908 is one of few non-JP2 genotype serotype b strains that has been analyzed previously by MLST (Haubek et al., 2007). This strain was in that study described as non-serotypeable. As HK908 in the study by Haubek and coworkers (Haubek et al., 2007) was used to illustrate the evolutionary stage, where non-JP2 genotype *A. actinomycetemcomitans* serotype b evolved into the JP2 genotype, we chose to include this strain also in the present study, after confirmation that it did in fact belong to serotype b (Johansson et al., 2017). For further comparisons, also the non-JP2 genotype serotype b strain, HK 912 was included as a reference. In the study by Haubek and coworkers (Haubek et al., 2007), HK908 was demonstrated to represent a breaking point, dividing JP2 and non-JP2 genotype strains of *A. actinomycetemcomitans* of serotype b into two different clusters. In this work, HK908 was instead used to illustrate clustering among non-JP2 genotype strains. As the deletion of an “A” at nt position 56 in *tbpA* was observed only in non-JP2 genotype serotype b strains in the previous study (Haubek et al., 2007), we here used the presence and the absence of this deletion as a means to divide the 56 non-JP2 genotype serotype b strains into two major clusters, A and B, respectively. This led to the observation that strains of Ghanaian and Swedish origin were differentially distributed in these clusters, again indicating that there is a genetic diversity between the two collections of non-JP2 genotype strains of *A. actinomycetemcomitans* serotype b from Ghana and Sweden, respectively.

Different types of PCR-based methods for the characterization of bacterial genomes have been developed during the last decades (van Belkum, 1994; Jolley and Maiden, 2014; Bonk et al., 2018; Wade and Prosdocimi, 2020). However, similar results will not necessarily be obtained, when bacterial collections are characterized with other PCR-based methods (Tsang et al., 2017). When we earlier characterized non-JP2 genotype strains of *A*. *actinomycetemcomitans*, serotype b by AP-PCR, we found more than 10 different banding patterns. The strains were grouped into AP-PCR types 1, 2 and others (referred to as AP-PCR type 3 in this work) (Claesson et al., 2017; Johansson et al., 2019). Among serotype b, strains of AP-PCR type 1 are in general more leukotoxic than the other AP-PCR types, and they are more frequently associated with periodontitis affecting young individuals (Dogan et al., 1999a; Höglund Åberg et al., 2014). Thus, albeit AP-PCR–based analyses of *A*. *actinomycetemcomitans* mainly reflects the general genetic diversity within this bacterial species, this methodology can be valuable for diagnostic purposes, to identify highly leukotoxic strains of serotype b. However, evidently AP-PCR is also a time-consuming method, which is associated with difficulties in standardization (Meunier and Grimont, 1993; Power, 1996; Tyler et al., 1997; Suzuki et al., 2003).

As evidenced from our present study, the MLST scheme implemented appeared not to be a highly discriminatory method to identify AP-PCR type 1, as strains of this AP-PCR type could be found within three different ST-types (ST 2, ST 3 and ST 88). Interestingly, AP-PCR type 1 A*. actinomycetemcomitans* strains of serotype b, isolated from both Ghana and Sweden, regardless being of JP2 or non-JP2 genotype, carry a gene that with very few known exceptions is present only in this AP-PCR type, i.e., *cagE* (Johansson et al., 2017; Johansson et al., 2019). As discussed earlier (Johansson et al., 2017; Johansson et al., 2019), the *cagE* gene product *per se* is most likely not responsible for the increased leukotoxicity of AP-PCR type 1 strains. However, to identify whether carriers are infected with a more virulent *A. actinomycetemcomitans* serotype b, i.e., AP-PCR type 1, which includes both JP2 and non-JP2 genotypes, detection of the *cagE* gene by PCR-based methodology appears to represent a more efficient tool than MLST.

## Conclusions

Although based on a limited number of assessed *A. actinomycetemcomitans* strains in this work, especially those collected from carriers in Sweden, there is a clear indication of a geographic dissemination across continents of non-JP2 genotype *A. actinomycetemcomitans* serotype b. Detection of the *cagE* gene is probably the currently known, most straightforward diagnostic procedure for detection of patients carrying the virulent AP-PCR type 1 of *A. actinomycetemcomitans*, serotype b. The *cagE* gene as a marker can be used to identify both JP2 and non-JP2 genotype strains of *A. actinomycetemcomitans* serotype b of this AP-PCR type, with PCR or qPCR, and *cagE*-specific oligonucleotide primers.

## Data Availability Statement

The original contributions presented in the study are included in the article/**Supplementary Material**. Further inquiries can be directed to the corresponding author.

## Author Contributions

Conceptualization, RC, AJ, and JO. Methodology, RC, AJ, AE, and JO. Validation, RC, AJ, CH, AE, and JO. Formal analysis, RC, AJ, and JO. Investigation, RC, AJ, and JO. Resources RC, AJ, CH, DH, and JO. Data curation, RC, AJ, and JO. Writing—original draft preparation, RC, AJ, and JO. Writing—review and editing, RC, AJ, CH, AE, DH, and JO. Visualization, RC, AJ, DH, and JO. Supervision, AJ, RC, and JO. Project administration, RC, AJ, and JO. funding acquisition, AJ and JO. All authors contributed to the article and approved the submitted version.

## Funding

This work was supported by TUA grants from the Region of Västerbotten, Sweden (AJ; 7003193 and JO; 7002667), and from Insamlingsstiftelsen, Medical Faculty, Umeå University, Sweden.

## Conflict of Interest

The authors declare that the research was conducted in the absence of any commercial or financial relationships that could be construed as a potential conflict of interest.

## Publisher’s Note

All claims expressed in this article are solely those of the authors and do not necessarily represent those of their affiliated organizations, or those of the publisher, the editors and the reviewers. Any product that may be evaluated in this article, or claim that may be made by its manufacturer, is not guaranteed or endorsed by the publisher.
