# Supplementary material for: Multilocus Sequence Typing of Non-JP2 Serotype b Aggregatibacter actinomycetemcomitans Strains of Ghanaian and Swedish Origin
Source: Front Cell Infect Microbiol. 2021 Dec 14;11:769671. doi: 10.3389/fcimb.2021.769671 (PMC8712761; doi:10.3389/fcimb.2021.769671)
Supplement: Supplementary file 1 [file DataSheet_1.pdf]

Table S1. List of all the 56 *A. actinomycetemcomitans* strains (all serotype b, and non-JP2 genotype), which were used in the present work. In total, 41 Ghanaian (Gh), and 13 Swedish (Sw) strains were assessed. HK908 and HK912 were included as reference *A. actinomycetemcomitans* strains. The AP-PCR type, sequence type (ST), and *cagE* genotype (+/-) is indicated for each strain. One strain was non-typeable (nt) by AP-PCR.

Strain number used in this work	Isolate number in the Ghanaian or Swedish collections	Name of strain	AP-PCR-type[1]	ST[2]	*cagE*[3]
Gh 01	001 Gh	062A	2	1	-
Gh 02	028 Gh	197A1	2	1	-
Gh 03	065 Gh	283A1	1	2	+
Gh 04	071 Gh	286A1	3	3	-
Gh 05	153 Gh	394A1	2	1	-
Gh 06	205 Gh	367A1	1	2	+
Gh 07	212 Gh	448A2	2	1	-
Gh 08	217 Gh	457A1	2	1	-
Gh 09	234 Gh	035A	3	3	-
Gh 10	369 Gh	180A	2	2	-
Gh 11	370 Gh	182C1	1	3	+
Gh 12	443 Gh	001A1	1	2	+
Gh 13	467 Gh	063A1	3	2	-
Gh 14	470 Gh	067A2	1	2	+
Gh 15	486 Gh	083A1	1	3	+
Gh 16	490 Gh	096A1	3	1	-
Gh 17	492 Gh	100A	2	5	-
Gh 18	493 Gh	136A1	2	1	-
Gh 19	519 Gh	158A	1	2	+
Gh 20	540 Gh	186A	2	1	-
Gh 21	575 Gh	204B1	1	2	+
Gh 22	581 Gh	208A1	1	2	+
Gh 23	585 Gh	220A1	3	2	-
Gh 24	589 Gh	284A1	1	2	+
Gh 25	605 Gh	299A1	1	2	+
Gh 26	619 Gh	372A1	nt	2	-
Gh 27	621 Gh	374A1	1	2	+
Gh 28	625 Gh	376A1	1	2	+
Gh 29	633 Gh	392A1	2	1	-
Gh 30	638 Gh	222A1	1	2	+
Gh 31	640 Gh	223A1	2	1	-
Gh 32	652 Gh	412A1	3	3	-
Gh 33	662 Gh	432A1	2	1	-

Gh 34	670 Gh	423A1	3	1	-
Gh 35	683 Gh	492A1	1	2	+
Gh 36	702 Gh	439A1	3	2	-
Gh 37	708 Gh	221A1	2	1	-
Gh 38	716 Gh	448A1	3	2	-
Gh 39	727 Gh	169A1	3	2	-
Gh 40	744 Gh	326A1	3	6	-
Gh 41	769 Gh	485A1	3	2	+
Sw 1	29 U	413-03	2	3	-
Sw 2	40 U	547-04	3	3	-
Sw 3	160 U	700-02	3	7	-
Sw 4	197 U	249-10	1	8	+
Sw 5	239 U	047-12	3	2	-
Sw 6	240 U	071-12	3	4	-
Sw 7	266 U	076-12	3	2	-
Sw 8	276 U	722-12	1	2	-
Sw 9	302 U	051-13	3	4	-
Sw 10	307 U	074-13	3	9	-
Sw 11	376 U	381-14	2	1	-
Sw 12	384 U	478-14	3	2	-
Sw 13	398 U	671-14	3	3	-
		HK908	2	3	-
		HK912	1	2	+

[1]Previously determined (Höglund Åberg et al., 2014; Claesson et al., 2017; Johansson et al., 2017; Johansson et al., 2019)

[2]Deduced in the present work by MLST as described in Materials and Methods

[3]Previously determined (Johansson et al., 2017; Johansson et al., 2019)

References

Claesson, R., Höglund-Åberg, C., Haubek, D., and Johansson, A. (2017). Age-related prevalence and characteristics of *Aggregatibacter actinomycetemcomitans* in periodontitis patients living in Sweden. *J Oral Microbiol* 9(1)**,** 1334504. doi: 10.1080/20002297.2017.1334504.

Höglund Åberg, C., Haubek, D., Kwamin, F., Johansson, A., and Claesson, R. (2014). Leukotoxic activity of *Aggregatibacter actinomycetemcomitans* and periodontal attachment loss. *PLoS One* 9(8)**,** e104095. doi: 10.1371/journal.pone.0104095.

Johansson, A., Claesson, R., Höglund Åberg, C., Haubek, D., and Oscarsson, J. (2017). The *cagE* gene sequence as a diagnostic marker to identify JP2 and non-JP2 highly leukotoxic *Aggregatibacter actinomycetemcomitans* serotype b strains. *J Periodontal Res* 52(5)**,** 903-912. doi: 10.1111/jre.12462.

Johansson, A., Claesson, R., Höglund Åberg, C., Haubek, D., Lindholm, M., Jasim, S., et al. (2019). Genetic profiling of *Aggregatibacter actinomycetemcomitans* serotype B isolated from periodontitis patients living in Sweden. *Pathogens* 8(3), 153. doi: 10.3390/pathogens8030153.
